# Exoscopic microvascular decompression for hemifacial spasm and trigeminal neuralgia

**DOI:** 10.3171/2023.10.FOCVID23122

**Published:** 2024-01-01

**Authors:** Hiroki Toda, Hirokuni Hashikata, Ryota Ishibashi

**Affiliations:** Department of Neurosurgery, Tazuke Kofukai Medical Research Institute Kitano Hospital, Osaka, Osaka, Japan

**Keywords:** exoscope, microvascular decompression, hemifacial spasm, trigeminal neuralgia

## Abstract

The 4K 3D exoscope system is becoming increasingly used in neurosurgery. Its 3D ultra-high-definition image is valuable in identifying and dissecting the delicate neural and vascular structures during microvascular decompression. In this video, the authors describe several nuances and details to perform the exoscopic microvascular decompression, including the exoscope layout and the modified supine position. Several illustrative case presentations highlight the benefits of exoscopic surgery. The authors’ exoscopic microvascular decompression series of 159 patients showed noninferior surgical outcomes compared to the operative microscope with no significant increase in surgical risk. In conclusion, an exoscope can be a practical alternative to performing microvascular decompression.

The video can be found here: https://stream.cadmore.media/r10.3171/2023.10.FOCVID23122

## Transcript

Exoscopes are becoming increasingly utilized in various neurosurgical procedures.^1–5^

### 0:26 Objective.

We describe nuances to perform the exoscopic microvascular decompression, including the exoscope layout and the patient position. Illustrative cases highlight the benefits of its 3D ultra-high-definition image in performing MVD (microvascular decompression). We also discuss the risk of using an exoscope for MVD. We obtained written informed consent from the patients.

### 0:49 Patient Population.

Since 2020, we have done over a hundred exoscopic MVD surgeries.

### 0:55 Exoscope Layout.

The exoscope layout is critical for obtaining the best surgical trajectory. We strongly advise placing the exoscope on the opposite side of the operation. With this setup, we can use the full range of motion of the exoscope arm joints and adjust the camera position finely. Furthermore, we place the monitor around 5 feet from the surgeon to achieve the best 3D effects.

### 1:21 Patient Position.

The exoscope can have various trajectory angles without significant stress to surgeons so that we can perform MVD in the supine position. We lift the patient’s ipsilateral shoulder, rotate the head to the contralateral side, and flex the neck slightly. Considering the intraoperative effects on venous and sinus pressure, we can slightly elevate the patient’s head. With this patient position, the patient’s shoulder does not interfere significantly with the surgeon’s procedure.

### 1:54 Retrosigmoid Approach.

During the approach, we place an exoscope camera above the head to record all surgical steps, such as preserving the lesser occipital nerve (LON), cutting the sternocleidomastoid (SCM) and splenius capitus (SpC) muscles, dissecting the mastoid emissary vein (MEV), exposing the mastoid notch (MN), coagulating and cutting the mastoid emissary veins around the mastoid foramen, and skeletonizing the mastoid foramen (MF).^6^ We cut the occipital artery (OA) and the styloid diaphragm to expose the inferior nuchal line (INL).

Then we detach the superior oblique capitus muscle (OCS) from the inferior nuchal line and expose the suboccipital surface inferiorly and laterally. We make a burr hole and a small bone flap using a steel burr and cutter. Using a rongeur and Kerrison punch, we expose the inferior sigmoid sinus and pack the mastoid air cells using a piece of bone wax. A dural incision releases CSF. After turning the dural flap, we can obtain a good trajectory for the cerebellomedullary cistern (CMCis).

### 3:33 Illustrative Case 1: Hemifacial Spasm, Gravity-Assisted Cerebellar Retraction.

We show an illustrative case of gravity-assisted cerebellar retraction in a right hemifacial spasm patient. After opening the cerebellomedullary fissure and additional arachnoid cutting, we notice that the upper loop of the PICA compresses the facial nerve root exit zone. We gently pull out the PICA, mobilize its upper loop, and decompress the root exit zone. Using a small amount of fibrin glue, we fix the PICA on the dural surface. We confirm decompression of the facial nerve root exit zone. Further mobilization of the AICA confirms the complete decompression of the facial nerve root. The patient has an immediate resolution of hemifacial spasm after the surgery.

During these procedures, the submonitor can show the operative view and the brainstem auditory evoked potentials side by side, which can provide us prompt feedback for hearing preservation.

### 4:35 Illustrative Case 2: Hemifacial Spasm, Perforating Artery and PTFE Fiber.

Another illustrative case highlights ultra-high-definition imaging of the AICA perforating arteries. The right AICA compresses the facial nerve root exit zone. The AICA loop has multiple perforating arteries behind the intermediate nerve (IMN). These perforating arteries limit the mobilization of the AICA. Therefore, we pull out the redundant AICA loop and use fine PTFE fibers to sling this loop.

There are two perforating arteries near the root exit zone. We use another PTFE fiber to mobilize and lift the main trunk of the AICA without jeopardizing the perforating artery. We fix the PTFE fibers on the petrous dura using fibrin glue and place the oxycellulose over the fibers. We confirm the complete decompression of the facial nerve root exit zone, and the patient experiences resolution of hemifacial spasm.

This PTFE sling technique is also proper when the perforating arteries are short, as seen in this left hemifacial spasm patient.

### 5:52 Illustrative Case 3: Hemifacial Spasm, Tortuous Vertebral Artery.

Next, we present a left hemifacial spasm patient with tortuous vertebral arteries. Both vertebral arteries deviate to the left. The right vertebral artery (Rt VA), VA union, and left vertebral artery (Lt VA) indirectly compress the facial nerve root. To make a space around the nerve root, we first glue the left VA to the right VA using a small drop of cyanoacrylate, press down the VA union, and fix it onto the dura using a fibrin-soaked TachoSil.^7^ After that, we pull the AICA out from the root exit zone and fix it onto the left VA using a fibrin-soaked TachoSil again. We confirm the decompression of the facial nerve root exit zone. The tortuous vertebral arteries are detached from the facial nerve, and the VA union remains attached to the dura at the level of the lower cranial nerves 12 months after the surgery. The patient has a gradual resolution of hemifacial spasm postoperatively.

A similar technique can mobilize a dolichoectatic VA, causing trigeminal neuralgia. We dissect the elongated VA from the lower cranial, facial, and auditory nerves. We move the VA downward, separate it from the trigeminal nerve, and fix it using fibrin-soaked TachoSil and cyanoacrylate. The 6-month postoperative images demonstrate the trigeminal and facial nerve decompression. The patient has been free from neuralgia.

### 7:38 Illustrative Case 4: Trigeminal Neuralgia, Arterial and Venous Compression.

The last case is trigeminal neuralgia with mixed arterial and venous compression. After the thorough arachnoid dissection,^8^ we mobilize and fix the superior cerebellar artery (SCA) on the tentorium using fibrin glue. Then we dissect the arachnoid around the transverse pontine vein (TPV) and coagulate and cut the vein. Then we mobilize and fix the vein of the cerebellopontine fissure (VoVPF) and cut the arachnoid around the Meckel’s cave to release the possible adhesion of the trigeminal nerve. The patient has immediate pain relief after surgery.

### 8:24 Outcomes and Complications.

Our exoscopic series outcomes do not differ from our previous microscopic series significantly in both hemifacial spasm and trigeminal neuralgia patients.

The recurrence rates in the present series remain low, but it may reflect the short follow-up period. Our exoscopic series has fewer transient facial and other cranial nerve dysfunctions and has several nonsevere adverse events. The slight difference between these two series may reflect the experiences of our surgical team.

### 9:01 Discussion on Pneumocephalus After Exoscopic MVD.

Significant pneumocephalus is a notable complication in exoscopic patients, as the supine position may induce spontaneous and sometimes excessive CSF drainage. To prevent this potentially hazardous complication, we tilt the operation table and fill the subdural space with plenty of artificial CSF just before tying the final dural sutures, which can reduce postoperative pneumocephalus.

### 9:29 Conclusions.

In conclusion, an exoscope can be an alternative to performing MVD, as it is not inferior to the operative microscope and can provide several clinical and educational benefits. The surgical team needs to optimize the operative layout and patient position. Significant pneumocephalus can be a hazardous complication, and we need to replace the lost CSF at the end of the procedure.

### 9:56 Acknowledgments.

The authors thank Namiko Nishida, Hideki Hayashi, Noriyoshi Takebe, Masahiro Sawada, So Matsukawa, Ryota Motoie, Kazushi Kitamura, Wataru Yoshizaki, Kazuhiro Kasashima, Jumpei Sugiyama, Takashi Hanyu, Masahito Yamashita, and Kazuya Ohtsuki for their surgical team organization and patient care and Koichi Iwasaki and Akinori Kondo for their supervision.

Thank you for your attention.

## Disclosures

The authors report no conflict of interest concerning the materials or methods used in this study or the findings specified in this publication.

## Author Contributions

Primary surgeon: Toda. Assistant surgeon: Hashikata, Ishibashi. Editing and drafting the video and abstract: Toda. Critically revising the work: all authors. Reviewed submitted version of the work: all authors. Approved the final version of the work on behalf of all authors: Toda. Supervision: Toda.

## Supplemental Information

### Patient Informed Consent

The necessary patient informed consent was obtained in this study.
